# Overcoming the Variability of iPSCs in the Manufacturing of Cell-Based Therapies

**DOI:** 10.3390/ijms242316929

**Published:** 2023-11-29

**Authors:** Suman C. Nath, Laura Menendez, Inbar Friedrich Ben-Nun

**Affiliations:** 1 Cell Therapy Process Department, Lonza Inc., Houston, TX 77047, USA; suman.nath@lonza.com (S.C.N.); laura.menendez@lonza.com (L.M.); 2 Cell Therapy Research and Development, Lonza Inc., Rockville, MD 20850, USA

**Keywords:** iPSC diversity, iPSC manufacturing, cell therapy, regenerative medicine

## Abstract

Various factors are known to contribute to the diversity of human induced pluripotent stem cells (hiPSCs). Among these are the donor’s genetic background and family history, the somatic cell source, the iPSC reprogramming method, and the culture system of choice. Moreover, variability is seen even in iPSC clones, generated in a single reprogramming event, where the donor, somatic cell type, and reprogramming platform are the same. The diversity seen in iPSC lines often translates to epigenetic differences, as well as to differences in the expansion rate, iPSC line culture robustness, and their ability to differentiate into specific cell types. As such, the diversity of iPSCs presents a hurdle to standardizing iPSC-based cell therapy manufacturing. In this review, we will expand on the various factors that impact iPSC diversity and the strategies and tools that could be taken by the industry to overcome the differences amongst various iPSC lines, therefore enabling robust and reproducible iPSC-based cell therapy manufacturing processes.

## 1. Introduction

**Figure 1:**
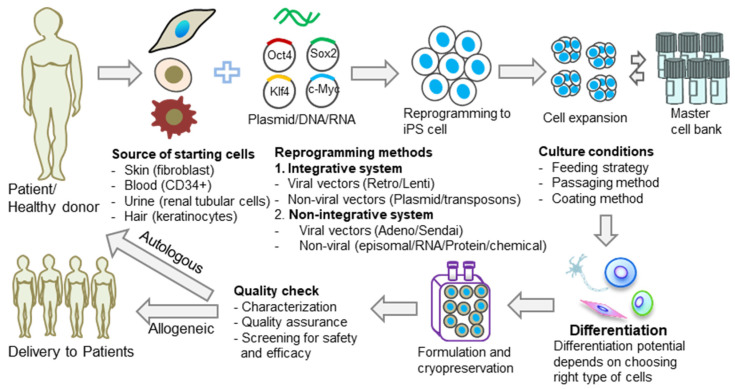
Diversity of iPSCs in cell therapy applications. Common causes of iPSC diversity are the source of the starting cells, reprogramming methods, and culture conditions. The effect of this diversity can, in turn, influence the differentiation potential of the generated iPSCs and, therefore, the final outcome of the therapy.

Induced pluripotent stem cells (iPSCs) are pluripotent stem cells made in vitro through the cellular reprogramming of somatic cells. iPSCs could be used for autologous cell therapy by reprogramming a patient’s own cells or for allogeneic cell therapy, which reprograms somatic cells that are obtained from healthy donors. iPSCs hold great promise in regenerative medicine and immune-oncology, and the genetic background of the somatic cells, along with the donor’s medical history, are critical attributes in ensuring the safety of iPSC-based therapies. At the moment, there is no standardization of the iPSC generation process. In contrast with the traditional clonal cell lines established, each iPSC line generated is different from other iPSC cell lines due to various characteristics, including the donor’s genetic background, the somatic cell type used for reprogramming, the reprogramming method, and the iPSC culture method (Figure 1). It should be expected, therefore, to find genomic and epigenomic variability when comparing different iPSC lines. This diversity could lead to differences in iPSC line stability in culture and differentiation potential and could, therefore, have a direct impact on the success of iPSC manufacturing processes.

### 1.1. Not All iPSC Lines Are Created Equally

#### 1.1.1. Donor Effect

Allogeneic iPSC-based cell therapy has the advantage of allowing donor screening for a variety of factors of interest. The donor’s genetic background and some epigenetic variations that could impact differentiation and the subsequent therapeutic potential are retained in the personalized iPSC line. Likewise, the disease-associated genetic variations found in donors could have an impact on the safety of the therapeutics, either by diminishing the therapeutic potential of the cells or by introducing mutated therapeutic cells into the patient. Kammers et al. performed a study with 194 healthy donors where 101 were female and 93 were male [1]. They isolated peripheral blood mononuclear cells (PBMCs) from the donors and reprogrammed them to iPSCs using *Oct4*, *Sox2*, *Klf4*, *c-Myc*, and *Lin28*. They then differentiated those iPSCs into megakaryocytes (MK). Transcriptomic profiling by RNA sequencing and proteomic profiling by mass spectrometry revealed variability in the CD41 and CD42a of MK expression across subjects. Numerous genes and protein expressions were different in subjects, dependent on sex and race, but, interestingly, not age. In another study, Yoshioka et al. found that by using four reprogramming factors of synthetic self-replicating RNA, the reprogramming efficiency of adult fibroblasts ended up lower than that of newborn fibroblasts [2]. The authors were able to increase the reprogramming efficiency of adult fibroblasts by adding an additional transcription factor to the reprogramming cocktail [3], implying that a reprogramming method might need optimization to successfully reprogram adult somatic cells. Therefore, the effect on iPSC diversity caused by the donor’s sex, race, and/or age might depend on the somatic cell type chosen.

In addition to the donor age’s impact on reprogramming efficiency, the age of the somatic cells and the stress they were exposed to before reprogramming also have an impact on the iPSC line genome integrity and overall iPSC quality. The donor’s family medical history also plays a role in donor screening; specifically in cases where the iPSC-based therapy is targeting cell/tissue replacement. For example, if the therapeutic target is an iPSC that will be differentiated to secrete insulin, it is important to choose a donor that has no mutations that would affect this pathway. In order to take advantage of donor variability to screen for a donor that matches certain criteria, it should be noted that the size of the donor pool is dependent on the desired somatic cell type to be reprogrammed. While the donor pool will be vast if fibroblasts or peripheral blood cells are to be reprogrammed, it is limited in size and availability if cord blood cells are to be reprogrammed.

#### 1.1.2. Somatic Cell Type

In the case of healthy donors, the younger the somatic cell type is, the lower the likelihood of it harboring pre-existing mutations. Cord blood CD34^+^ cells, for example, are acquired at birth from the umbilical cord. Those cells are, therefore, young, and exhibit stem cell characteristics, which make them an excellent somatic cell type for cellular reprogramming. On the other hand, the availability and donor pool size are limited for the acquisition of cord blood cells. Other, more accessible, somatic cell types, such as PBMCs and fibroblasts obtained from skin biopsy, have been successfully reprogrammed to iPSCs. Skin fibroblast was the first somatic cell type to be reprogrammed to iPSCs by Takahashi and Yamanaka [4]. Since then, it has been shown that fibroblasts can be isolated from different organs and tissues for reprogramming, e.g., dermal, lung, cardiac, or periodontal ligaments [5,6,7,8]. Along with skin fibroblasts and cord blood cells, other accessible starter cells for reprogramming are peripheral blood cells [9], exfoliated renal tubular epithelial cells obtained from urine [10], and keratinocytes from plucked hair [11]. It has been claimed that, among these starter cells, renal tubular epithelial cells and keratinocytes are superior in their accessibility and ease of isolation [12]. The reprogramming efficiency is also higher for these two cell types and requires relatively less time for reprogramming [11,12]. Nevertheless, the somatic cell types that are most currently used by therapeutic companies to generate iPSCs are fibroblasts and blood cells. 

Along with the donor screening described above, testing the specific somatic cell type to be reprogrammed for genetic mutations is crucial to ensure the safety of therapeutics [13,14,15]. Skin fibroblasts are more prone to acquire additional DNA damage compared to blood cells as a result of the continuous exposure of skin to environmental stress, such as UV irradiation. This was shown in a study by Rouhani et al. which compared 696 hiPSCs and daughter subclones [16]. They showed that about 72% of iPSCs had genomic heterogeneity due to UV-related damage, confirming that iPSCs derived from skin fibroblast cells harbor more pre-existing genetic mutations compared to blood-derived iPSCs. Also, is important to note that, in general, the given somatic cell type to be reprogrammed will dictate the respective reprogramming method and vice versa (see section below for more details). 

#### 1.1.3. Reprogramming Method

Various methods of cellular reprogramming exist, and they have been used to reprogram various somatic cell types into pluripotent stem cells. The reprogramming methods can be divided into two major groups: integrative and non-integrative transfer systems [17] (Figure 1). The integrative transfer system consists of viral vectors (retrovirus or lentivirus) and non-viral vectors (plasmid or transposons, e.g., PiggyBac/Sleeping Beauty) that induce the integration of the transgene into the host cell genome. The non-integrative transfer system also consists of viral vectors (adenovirus or Sendai virus) and non-viral vectors (episomal, protein, RNA, or microRNA) that deliver the transgene into the host cells and enable the temporal, non-constitutive expression and activation of the transgene in the host cell. The first iPSCs were generated by the Yamanaka group using the integrative transfer system with lentiviral vectors [4]. Among the viral vectors, lentiviral vectors are superior to retroviral vectors in iPSC reprogramming because of their broad tropism and reprogramming efficiency [18,19]. The integration of transgenes into the host cell genome poses, however, the risk of mutagenesis and genome aberration as a result of random, uncontrolled genome integration. In some cases, this might necessitate the removal of exogenous transgenes from the cells after reprogramming is achieved. Such techniques could be combined with the non-viral transfer system that uses a mobile genetic element, e.g., PiggyBac transposons [20,21,22]. However, applying these techniques for iPSC reprogramming yields low reprogramming efficiency and leads to the risk of reintegration [23]. Therefore, the non-integrative transfer system is most desirable for iPSC reprogramming in cell therapy applications. Adenovirus and Sendai virus-mediated vector delivery provide alternative non-integrative methods for iPSC reprogramming [24,25,26,27,28]. Although both methods have low reprogramming efficiency, Sendai virus-mediated reprogramming has been used for several clinical applications, notably, in cystic fibrosis, the AIDS vaccine, and other iPSC replacement therapies [29,30,31,32,33]. Another example of a non-integrative cellular reprogramming technique is episomal-based reprogramming, which relies on a one-time introduction of episomal DNA plasmid vectors encoding reprogramming-inducing transcription factors [34,35,36]. The episomal DNA vectors also encode for the EBNA-1 protein, which enables them to persist in the cells longer than transient DNA vectors. Reprogramming with episomal DNA vectors could be applied to any somatic cell type, although it often requires different transfection methods to be applied to each cell type. The efficiency of the episomal-based reprogramming method and the length of the reprogramming process are, therefore, variable, and dependent on the somatic cell type [34,35,36]. While episomal-based reprogramming is considered a “zero-footprint” method, as this method involves DNA plasmid, there is a need to keep the generated iPSC colonies in culture long enough to allow for the vector to be eliminated through the process of cell division (vector clearance). Vector elimination is tested through sensitive analytical methods such as PCR. As opposed to reprogramming by episomal DNA vectors, mRNA-based reprogramming relies on sequential introductions of mRNA sequences encoding for reprogramming inducing transcription factors [37]. While selected iPSC colonies could be expanded immediately, as the mRNA half-life is short due to its nature, mRNA reprogramming is usually applied only to cells that can survive multiple rounds of transfections, such as adherent cells (i.e., fibroblasts). In other words, it cannot be applied successfully to cells that are inherently grown in suspension (such as blood cells) due to the negative impact of multiple rounds of transfections on cell viability. To circumvent this problem, one group published the derivation of endothelial progenitor cells from PBMNCs, followed by reprogramming this cell population to iPSCs by mRNA reprogramming [38]. This method, however, adds time and labor to the reprogramming process and there are no additional publications showing it could be applied to blood cells of other sources (i.e., cord blood). Another method for reprogramming relies on the delivery of the transcription factors in their protein form [39,40,41]. This method has the drawback of low reprogramming efficiency compared to other reprogramming methods. Chemical programming is an attractive “zero-footprint” reprogramming method for clinical-grade iPSC generation [42,43,44]; however, most of the chemical reprogramming methods developed so far have used mouse cells because of their stable epigenome and high plasticity compared to human cells [45,46,47,48]. The Hongkui group recently succeeded in generating iPSC from human somatic cells using the chemical reprogramming method [49]. Unlike mouse cells, reprogramming human somatic cells by chemical reprogramming required the transformation of the somatic cells into an intermediate state, prone to reprogramming, and then the reprogramming was induced. This reprogramming method, however, took 50 days and had a reprogramming efficiency of 0.2–2.5%. The same group systematically optimized the original protocol by adding small molecule boosters to enhance the reprogramming efficiency, which reduced the reprogramming time from 50 days to 16 days [50].

#### 1.1.4. Somatic Cell Lineage and Differentiation Commitment

Somatic cells, and even somatic stem cells, are lineage-committed. While cellular reprogramming reverts to the differentiated state of the cells, epigenetic memory and lineage-specific DNA methylation marks could affect the differentiation potential of the generated iPSC line [51,52,53]. Although iPSCs are pluripotent by definition, and, therefore, are capable of giving rise to cells of the three germ lines, epigenetic differences could necessitate further optimization of a differentiation protocol for the specific iPSC line. With the advancements in cell selection and purification, acquiring somatic cells for iPSC generation of the same lineage or even cell type as the intended therapeutic cell type is possible in some cases. For example, for some immune-oncology applications, the reprogramming of T cells with a specific T cell receptor (TCR) rearrangement could be a great advantage in order to have the desired TCR in the iPSC line.

The type of starting cells for reprogramming also determines the commitment of the differentiation capabilities of the generated iPSCs. For example, iPSCs derived from human mesenchymal stem cells (hMSCs) have higher capabilities of differentiation into cardiomyocytes compared to iPSCs derived from keratinocytes and skin fibroblasts [54]. This is because cardiomyocytes are mesodermal in origin, and as hMSCs originate from a population of mesodermal cells, they contain the epigenetic memories to convert into cardiomyocytes more efficiently. Therefore, when possible, considering the right type of starting cells for reprogramming is important to achieve the targeted outcome of the iPSC-derived final cell therapy product.

#### 1.1.5. Culture Conditions

The culture conditions could also have an impact on iPSC diversity. Several studies comparing various iPSC media, along with the passaging method and feeding strategy, have shown their effect on the iPSC culture morphology and expansion rate. Although the mutational burden comes mostly from starting cells, the cell culture, passaging reagents, and passaging method could impact the mutational rate [13,14,15,54,55]. Laurent et al. performed a study with 186 pluripotent and 119 non-pluripotent cells and analyzed changes in the copy number of chromosomes. The study determined that reprogramming methods can remove the tumor suppressor genes from cells, and culture time can trigger the duplications of oncogenic genes in iPSC [13]. Mayshar et al. also reported that reprogramming methods and culture adaptations are responsible for the chromosomal aberrations in iPSCs [15]. They also reported chromosome 12 duplications with the upregulation of NANOG, which is a pluripotency marker. The methods for coating and passaging also contribute to changing the properties of iPSCs. In a long-term study with more than 100 passages of iPSCs, a group of researchers found that enzymatic passaging caused a higher growth rate, and more genetic instability and OCT4-positive cells in teratoma compared to mechanical passaging [54]. Similar results were also observed when comparing iPSCs cultured in feeder-free versus feeder-dependent (MEF, mouse embryonic feeder) conditions. Moreover, the negative impact of enzymatic versus mechanical passaging was also observed in iPSC cultures with an MEF layer. Therefore, the culture conditions create heterogeneity in iPSC expansion which ultimately results in creating iPSC diversity. 

### 1.2. Diversity of Equally Created iPSC Lines

As summarized above, some of the most critical effectors in determining the performance of the generated iPSC line are the donor, the somatic cell type, the reprogramming method, and the culture platform. Keeping these critical effectors the same should standardize iPSC manufacturing. Nevertheless, keeping the above parameters the same still results in iPSC colonies that are different from each other. Reprogramming, for example, a given somatic cell type from a given donor, using a given reprogramming method and culture platform, may give rise to iPSC colonies that are different in their expansion rate, robustness (the tendency to spontaneously differentiate in culture), and potential to be differentiated directly to specific lineages [56,57,58,59,60]. Interclonal variability can also arise from genetic mutations occurring during culture [13]. It can also arise from alterations in DNA methylation during reprogramming or culture [61,62,63]. Although the variability amongst different clones from the same donor is lower than the variability of iPSCs generated from different donors, it still leads to a vast iPSC line diversity, which causes numerous hurdles in the standardization of iPSC manufacturing processes. For example, it results in the selection of a large number of iPSC clones, followed by expansion through passaging to establish robust cell lines. Thus, the successfully cultured iPSC lines are then tested for differentiation capability in order to choose the ‘golden’ iPSC line for the specific cell therapy product. 

### 1.3. Methods and Platforms to Standardize iPSC Manufacturing Processes

As with any successful manufacturing process, hiPSC manufacturing necessitates robust and reproducible methods and protocols. As described above, the reprogramming efficiency is dependent on the donor’s age, the somatic cell type, and the reprogramming method. Likewise, whether it is an iPSC line that one has established or acquired, differences between iPSC lines are inevitable and those differences pose a risk to the manufacturing process and contribute to incidences of process deviation and even process failure. At the end of 2021, Kim et al. cited that from the 19 therapeutic trials using hiPSC that had been published at that moment, 15 (79%) were allogeneic and 4 were autologous therapies. For those four autologous trials, differences during the differentiation process of those iPSC lines would have a major impact on the cost and cause a higher probability of deviations during the manufacturing process.

To mitigate these risks, there are several solutions and best practices that could be taken to standardize and harmonize iPSC manufacturing, strategies that are, especially, focused on increasing process quality and comparability. Since the discovery of iPSC generation, the field has transitioned from using PSC culture media containing animal reagents to Xeno-free and even animal-free media [64,65,66]. The use of defined culture media with recombinant proteins instead of whole animal serum greatly increases process comparability and supply chain assurance [67,68,69]. 

**Figure 2:**
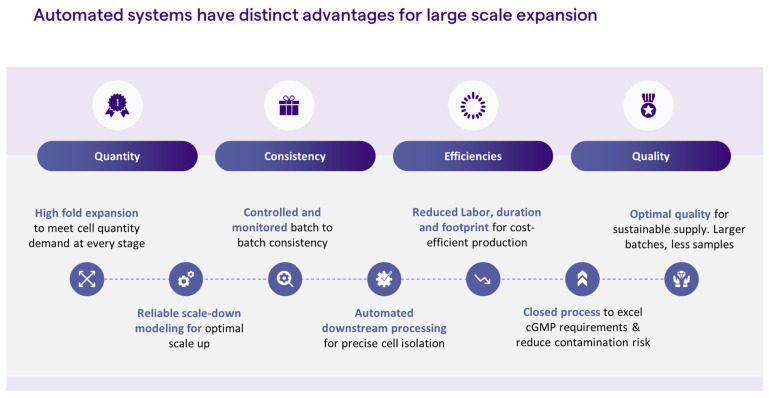
Advantages of automated systems.

iPSC reprogramming and expansion is a highly manual process, and, as such, is governed by subjective decision-making. The operator makes a subjective decision as to which iPSC colony to pick, usually based on colony morphology. The colony is then picked manually by the operator who practices subjective decision-making each time cell passaging is employed, through an evaluation of culture confluency, morphology, and/or percentage of spontaneous differentiation These cell culture processes are not only highly manual but call for highly trained personnel. Replacing manual steps with automation could greatly contribute to process consistency and product quality. Coupled with parameter monitoring, process automation for feeding and passaging would enable unbiased decision-making for the delivery of high-quality cells. Moreover, automation enables process scale-up, therefore, meeting the large quantities that are needed for cell therapy indications, such as immunotherapy and regenerative medicine, while reducing labor, footprint risk of safety issues, and, ultimately, cost (Figure 2). Below, we discuss some strategies for introducing automation into the iPSC manufacturing process.

#### 1.3.1. Automated iPSC Handling

Since, for the most part, iPSC generation, expansion, and differentiation are manual processes, iPSC-derived therapies are labor-intensive and time-consuming and may not be a feasible option for commercial manufacturing. Coupled with the high variability of iPSC generation, it makes the automation of the process a great alternative to provide more robust and straightforward manufacturing for cell therapy applications. Several models of automation of iPSC generation, expansion, and differentiation have been demonstrated in recent years [70,71,72,73,74,75,76]. Paull et al. reported the development of a robotic platform for the reprogramming and expansion of iPSCs from skin biopsies and successfully differentiated the cells with minimal manual intervention [70]. They automated the isolation of skin fibroblasts from 640 skin tissue samples and made a bank of fibroblasts at earlier passage using liquid handling automation. They also applied the robotic platform for the thawing and seeding of fibroblasts, adding reprogramming cocktails, and the isolation of nascent iPSC colonies followed by automated differentiation. Similarly, Bando et al. reported the development of an automated machine for the efficient expansion of iPSCs which could be differentiated into cardiomyocytes, hepatocytes, neural progenitors, and keratinocytes [72]. Konagaya et al. maintained hiPSCs for 60 days using their developed automated system which could be differentiated into all three germ layers [74]. Tristan et al. also reported the expansion and differentiation of 90 different patient/disease-specific cell lines using a robotic platform [75]. Elanzew et al. developed StemCellFactory, a modular system that integrates the generation and expansion of iPSCs automatically in 24-well plates or scale-up in 6-well plates [76]. However, several studies reported that the development of automated systems in 2D and 3D systems has remained unexplored for the generation, expansion, and differentiation of iPSCs. Industrial manufacturing is not yet in full bloom for adapting an automated system that combines all steps of iPSC generation, expansion, and differentiation. 

#### 1.3.2. Moving away from 2D Cell Culture to 3D Cell Culture

The benefits of moving away from a conventional 2D culture to a 3D suspension culture platform are clear. The use of 3D culture platforms, such as bioreactors, enables the process to be controlled, monitored, closed, and automated, therefore greatly contributing to cGMP compatibility for manufacturing both autologous and allogenic therapies. Three-dimensional bioreactors are closed compartments, and the process steps of media changing and cell seeding, expansion, passaging, and harvest could be performed in a closed manner. Likewise, those platforms are scalable, contributing, therefore, to the clinical and commercial viability of the cell therapy product. It has been shown that iPSCs can be expanded in suspension either as aggregates [77,78] or on microcarriers [79,80]. While culturing iPSCs as aggregates involves 2D to 3D adaptation and frequent cell passaging to control the size of the cell aggregates, the cells could be directly used for downstream processes such as cryopreservation and differentiation [79,81]. Culturing iPSCs in a stirred tank bioreactor on microcarriers could be an attractive way to achieve large cell quantities in a relatively short time, without the need for 2D to 3D adaptation or passaging [81]. An end-to-end process of thawing and expanding iPSCs in a stirred tank bioreactor was recently described [81]. In this process, iPSCs were cultured in agitation, on microcarriers, with no passaging for 12–14 days, followed by steps of dissociation from the microcarriers, microcarrier removal, and cell concentration, all prepared in a closed manner. The harvested cells could then be either cryopreserved or directly differentiated. In addition, a 3D seed train was enabled, either by re-inoculating single cells post-harvest with microcarriers or by transferring the cells attached to microcarriers to a bioreactor with microcarriers. Moreover, it was shown that the initial step of expanding iPSCs in 2D, in order to reach the required cell number to initiate a bioreactor culture, could be omitted from the process by directly thawing cryopreserved iPSCs into the bioreactor. This would greatly improve process comparability and reproducibility by completely avoiding a 2D cell culture, which is highly manual and subjected to biased decision-making as to culture healthiness and confluency which contributes to process deviation. 

#### 1.3.3. Analytics

Cell release characterization assays are a key tool to ensure cell identification, safety, and the potency of cell therapy intermediate and end products. In the iPSC manufacturing process, those assays are pivotal and should be performed along the various steps of the process, including reprogramming, expansion, cryopreservation, and differentiation. The detailed characterization of human iPSCs manufactured for therapeutic applications, as described by Baghbaderani et al., includes release assays to determine the cell identity, purity, and safety (i.e., cell count and viability, pluripotency markers, karyotyping, mycoplasma, sterility, and endotoxin and viral testing), while characterization assays (i.e., embryoid body formation, gene array analysis, morphology, and post-thaw plating) show the cell morphology, genotyping, and the ability of the cells to differentiate [82]. Babu et al. also emphasized the need for a release assay which forms the basis of process parameters and critical quality attributes [83]. They reported that the automation of release assays could deliver the iPSC-derived cell therapy products in a robust and reproducible manner.

## 2. Conclusions

Diversity is a widely known characteristic between different iPSC lines and even amongst clones from the same lines. Donor-specific genomic attributes, as well as the epigenetic attributes of the specific somatic cell type, are the hallmarks of iPSC diversity. As such, the omics of a given iPSC line are highly determined and affected by the omics of the donor somatic cell type. This diversity of iPSC lines can be beneficial when used to generate a library of differentiated cell types (i.e., hepatocytes) of various donors for identifying ethnic-specific drug side effects, improving, for example, drug safety [84]. Also, a well-characterized iPSC bank with diverse iPSC lines could work as a useful resource, demonstrating a normalized control for in vitro human development and disease modeling [85]. On the other hand, the diversity of iPSC lines is a major disadvantage in the standardization of cell manufacturing for therapies using iPSCs as a source. The screening of iPSC lines to identify lines with better yield and differentiation potential is required before mass production, and standardization efforts are necessary for the analytical methods (either cell-based or molecular assay) required for iPSC identification and the indication of their functionality.
